# Association between ultra-processed food consumption and perceived stress among teaching professionals: a gender-stratified cross-sectional study from Urban India

**DOI:** 10.3389/fnut.2026.1889456

**Published:** 2026-08-21

**Authors:** Anindita Ghosh, Arti Muley, Reena Verma, Ashutosh Zunjur, Saylee Borse, Subhamitra Adhikari

**Affiliations:** 1 Department of Nutrition and Dietetics, Symbiosis Skills and Professional University, Pune, Maharashtra, India; 2 Department of Nutrition and Dietetics, Symbiosis School of Culinary Arts and Nutritional Sciences Symbiosis International (Deemed University), Pune, Maharashtra, India; 3 School of Ports and Logistics and School of Interdisciplinary Science, Symbiosis Skills and Professional University, Pune, Maharashtra, India

**Keywords:** cross-sectional study, dietary behavior, gender differences, NOVA classification, perceived stress, PSS-10, teaching professionals, ultra-processed food

## Abstract

**Background:**

Ultra-processed foods (UPFs) have become deeply embedded in contemporary dietary patterns, particularly among urban working populations. Concurrently, perceived stress especially in high-demand occupations such as teaching has emerged as a significant public health concern. Understanding the relationship between UPF consumption and perceived stress is essential for designing targeted nutritional and mental health interventions.

**Objective:**

This study investigated the association between ultra-processed food consumption and perceived stress levels among teaching professionals, with specific attention to gender-related differences in dietary behavior and stress experience.

**Methods:**

A cross-sectional analytical study was conducted among 549 teaching professionals from educational institutions across urban India. UPF consumption was quantified using a validated 20-item binary-scored instrument aligned with the NOVA classification system. Participants were stratified into Low ( ≤ 8 items; *n* = 149), Moderate (9–12 items; *n* = 241), and High (≥13 items; *n* = 159) UPF consumption groups. Perceived stress was assessed using the validated 10-item Perceived Stress Scale (PSS-10). Analytical methods included Welch-corrected independent samples *t*-tests, Pearson and Spearman correlation analyses, simple and multivariable linear regression.

**Results:**

Mean PSS-10 scores increased progressively across Low (2.77 ± 0.54), Moderate (3.01 ± 0.36), and High (2.97 ± 0.32) UPF consumption groups. Welch-corrected *t*-test demonstrated significantly higher perceived stress in the High versus Low group [t_(236.67)_ = −3.95, *p* < 0.001; Cohen's *d* = 0.70). Pearson correlation revealed a significant positive association (*r* = 0.210, *p* < 0.001; R^2^ = 0.044), confirmed by Spearman's ρ = 0.178 (*p* < 0.001). Multivariable regression showed UPF consumption remained significantly associated with perceived stress after adjustment for age, gender, and BMI (β = 0.175, *p* = 0.003). Female teachers reported significantly higher perceived stress than males across all UPF strata (β = 1.42 PSS points, *p* = 0.020).

**Conclusion:**

Higher UPF consumption was significantly but modestly associated with elevated perceived stress among teaching professionals. The observed association is consistent with mechanistic pathways described in prior experimental literature, including gut-brain axis disruption and neuroendocrine dysregulation; however, these mechanisms were not empirically tested in the present study. Female educators demonstrated disproportionately higher stress burden. Findings support incorporating dietary behavior as a modifiable target within occupational wellness strategies for educators. Longitudinal, biomarker-inclusive, and gender-stratified research is warranted to establish directionality, test inflammatory and gut-brain mechanisms, and develop evidence-based occupational wellness interventions.

## 1 Introduction

Perceived stress, defined as an individual's subjective appraisal of the extent to which life circumstances feel uncontrollable, unpredictable, and overwhelming, has emerged as a central construct in both clinical and public health research (1). Unlike objective stressor-based models, this construct captures inter-individual variation in how environmental demands are interpreted and emotionally processed, making it particularly sensitive to contextual, personality-related, and social determinants (2). Individuals confronting ostensibly equivalent stressors may report markedly divergent levels of perceived stress, depending on coping repertoires, social support networks, and structural circumstances.

From a gender perspective, perceived stress is shaped by socially constructed roles and institutional inequities. Women in professional occupations frequently shoulder a dual burden of paid work and unpaid caregiving, navigating gendered performance expectations and limited institutional accommodation-factors that independently amplify perceived stress beyond occupational demands *per se* (3, 4). This gendered dimension necessitates analytical approaches that move beyond aggregate associations toward gender-stratified inquiry.

Teaching professionals represent a particularly salient population for such inquiry. The occupation has undergone progressive intensification, with educators contending with escalating administrative pressures, rapid technological transitions, performance accountability mandates, and chronic work–life imbalance (5). These occupational demands carry well-documented consequences, including burnout, diminished instructional effectiveness, psychological distress, and early attrition. Female teachers face compounded vulnerabilities from emotional labor demands, caregiving obligations, and precarious contractual arrangements, particularly in private educational settings.

The epidemiological burden of stress among educators is considerable and globally distributed. A scoping review identified stress prevalence ranging from 8.3% to 87.1% across diverse national contexts (5). The COVID-19 pandemic further intensified this burden, with nearly 30% of teachers globally reporting significantly elevated stress during enforced transitions to remote pedagogy (6). Region-specific data are equally striking: 50.1% of Ethiopian primary school teachers experienced occupational stress during the second COVID-19 wave, and 26.3% of Canadian teachers across three provinces reported high perceived stress (7, 8).

Concurrently, ultra-processed food (UPF) consumption has increased substantially in urban populations, including India. UPFs are industrially formulated products characterized by multiple processing stages, incorporation of food-derived additives, and inclusion of substances rarely used in domestic cooking such as artificial flavors, emulsifiers, sweeteners, and preservatives primarily engineered for palatability, convenience, and extended shelf life (9, 10). In urban India, UPFs now contribute meaningfully to total daily energy intake among adolescents and working adults, reflecting a rapid dietary transition toward processed food consumption (11–13).

A growing body of evidence associates habitual UPF consumption with adverse health outcomes spanning cardiovascular disease, hypertension, obesity, and type 2 diabetes, gastrointestinal disorders, mental health conditions including depression and anxiety, and all-cause mortality (14–19). An umbrella review of epidemiological meta-analyses encompassing nearly 10 million participants confirmed consistent harmful associations across multiple disease categories (20). Mechanistically, multiple biological pathways link UPF consumption to psychological stress: chronic low-grade inflammation activating the hypothalamic-pituitary-adrenal (HPA) axis; emulsifier- and sweetener-driven disruption of gut microbiota composition affecting the gut-brain axis; neuroactive additives modulating serotonin and dopaminergic systems; and high-glycaemic dietary patterns generating recurrent cortisol surges (16, 21, 22).

The NOVA classification system provides the methodological framework most widely employed in UPF research, distinguishing four food groups by the nature, extent, and purpose of industrial processing (9, 23).This framework enables standardized, cross-study comparisons validated across numerous epidemiological contexts.

**Figure 1:**
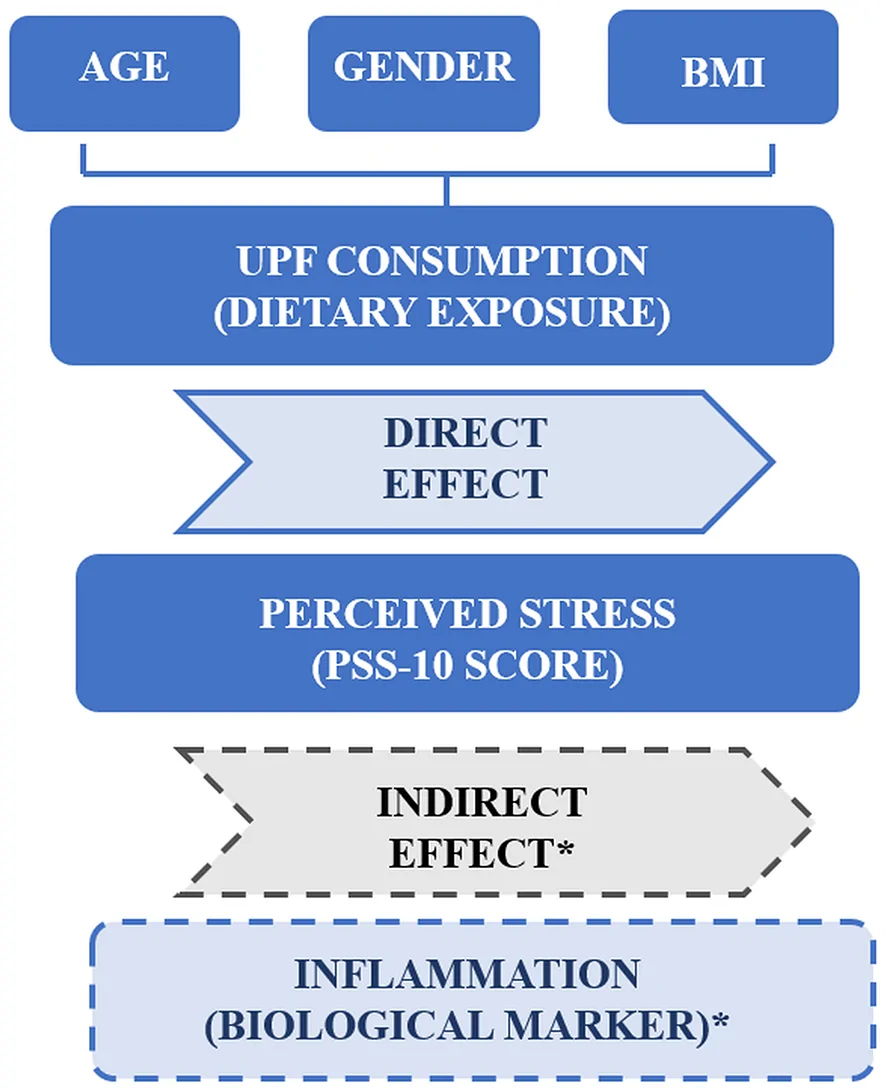
Conceptual framework: UPF consumption and perceived stress among teaching professionals. The model illustrates bidirectional pathways between occupational stress, gender, and dietary behavior mediated by inflammatory, neuroendocrine, and gut-brain axis mechanisms. *Hypothesized pathway–inflammatory markers were not directly measured in the present study. The indirect effect via inflammation represents a proposed biological mechanism and was not empirically tested.

Despite the convergence of occupational stress, gendered vulnerability, and rising UPF consumption in the Indian teaching workforce, the specific relationship between UPF intake and perceived stress in this population remains empirically underexplored. Moreover, stress may itself function as an antecedent of UPF consumption, insofar as psychological distress predisposes individuals toward energy-dense, convenient dietary choices as a maladaptive coping strategy-a dynamic potentially more pronounced among female educators given time scarcity and caregiving constraints. Figure 1 depicts the conceptual framework depicting the association between UPF consumption and perceived stress among urban teaching professionals. Solid elements represent pathways empirically examined in the present study. Dashed elements indicate a hypothesized indirect pathway via inflammation; no inflammatory biomarkers were directly measured in the present study. The inflammatory pathway depicted in Figure 1 represents a hypothesized biological mechanism drawn from existing literature and was not directly examined in the present study, as no inflammatory biomarkers were measured.

The present study was therefore designed to examine the association between ultra-processed food consumption and perceived stress levels among teaching professionals, employing validated instruments within a cross-sectional analytical framework. Findings are intended to inform gender-responsive dietary and mental health interventions within the Indian educational sector.

## 2 Materials and methods

### 2.1 Study design and setting

A cross-sectional analytical study was conducted between September and December 2025 across selected educational institutions and community centers in Pune, Maharashtra, India. This design enables simultaneous assessment of exposure (UPF consumption) and outcome (perceived stress) variables within a single timeframe, supporting hypothesis generation for future longitudinal investigation. The study is reported in accordance with the Strengthening the Reporting of Observational Studies in Epidemiology (STROBE) checklist for cross-sectional studies; a complete checklist is provided as Supplementary File 1.

### 2.2 Ethical approval and participant consent

Ethical clearance was obtained from the Institutional Review Board (IRB) of Symbiosis Skills and Professional University, Pune, prior to data collection commencement. The study was conducted in full compliance with the principles outlined in the Declaration of Helsinki (2013 revision). All participants received comprehensive information regarding study objectives, procedures, confidentiality safeguards, and their unconditional right to withdraw without consequence. Written informed consent was obtained from each participant prior to enrolment. No personally identifiable data were retained beyond the study period.

### 2.3 Participants and sampling strategy

Convenience sampling was employed to recruit 549 teaching professionals aged 18–60 years from educational institutions across the study region. Inclusion criteria required participants to be currently employed as teaching professionals; fall within the specified age range; be capable of independently completing structured questionnaires; and provide voluntary written informed consent (13). Questionnaires were distributed to 631 teaching professionals across 14 educational institutions in Pune during September–December 2025. Of these, 82 were excluded prior to analysis: 34 returned incomplete questionnaires (>20% missing items), 26 did not meet inclusion criteria (non-teaching administrative roles, *n* = 18; age outside the specified range, *n* = 8), and 22 declined participations after receiving study information. The final analytic sample comprised 549 participants (response rate: 87.0%). Item-level missingness among the 549 retained participants was minimal (< 1.2% of cells across all variables). The age distribution (78.5% aged 20–39 years) and gender composition (52.3% male) of the present sample are broadly consistent with the demographic profile of urban private-sector teaching professionals reported in the National Sample Survey (2022–23 education round), lending contextual plausibility to the sample composition despite the non-probability sampling design. Exclusion criteria comprised: current diagnosis of an eating disorder; severe mental health condition precluding reliable self-report; inability to provide informed consent; or incomplete questionnaire responses (>20% missing items). The target sample size was pre-specified to achieve 80% statistical power for detection of a moderate effect size (*r* = 0.15) at a two-tailed significance threshold of α = 0.05, requiring a minimum of 348 participants. The achieved sample of 549 provided greater than 95% power.

### 2.4 Assessment of ultra-processed food consumption

Ultra-processed food consumption was assessed using a 20-item researcher-developed checklist derived from NOVA Group 4 food categories (9, 24) and adapted to reflect the Indian urban dietary context (13). Items were drawn from food groups well-documented in the Indian UPF literature and include packaged snack foods, carbonated beverages, instant noodles, ready-to-eat meals, flavored dairy products, and commercially produced baked goods. Content validity was established through review by a panel of three registered nutrition professionals prior to administration; minor revisions were made following this review. The checklist was piloted in 30 urban teaching professionals from the same region; no items required removal. Cronbach's alpha was not computed, as this is a frequency checklist assessing dietary variety rather than a psychometric scale measuring a latent construct; internal consistency is not a meaningful metric for such an instrument.

Each item was coded as 1 (consumed) or 0 (not consumed) during the reference period. Scores ranged from 0–20, with higher scores indicating greater UPF exposure. The UPF score was used as the primary exposure variable in all inferential analyses. Twenty-four-hour dietary recall data were collected only for descriptive dietary characterization and were not included in regression analyses. The relationship between occupational contexts and UPF consumption has been further documented in recent employee-based studies supporting the relevance of this dietary dimension in working professional populations (24).

### 2.5 Assessment of perceived stress

Perceived stress was measured using the 10-item Perceived Stress Scale (PSS-10), a widely validated instrument that assesses the degree to which individuals appraise their life circumstances as stressful over the preceding month (1, 8, 25).Each item is rated on a five-point Likert scale (0 = Never to 4 = Very Often), with items 4, 5, 7, and 8 reverse-scored per standard protocol. The PSS-10 demonstrates good internal consistency (Cronbach's α = 0.78–0.91), strong test-retest reliability (*r* > 0.70), and well-established construct validity across diverse populations, including South Asian cohorts (26).

### 2.6 Statistical analysis

Statistical analyses were performed using R (version 4.3.1). Descriptive statistics were computed for all continuous variables. Normality was assessed using Shapiro–Wilk tests and Q–Q plots; outliers were identified using the interquartile range criterion. Given confirmed heterogeneity of variance (Levene's test: *F* = 26.82, *p* < 0.001), Welch-corrected independent samples *t*-tests were applied for group comparisons. Pearson correlation assessed linear associations between continuous UPF scores and PSS-10 scores; Spearman's ρ served as a non-parametric sensitivity check. Effect sizes were reported using Cohen's d for group comparisons and standardized β for regression models.

Simple and multivariable linear regression models were constructed, with Model 2 adjusting for age, gender, and BMI. The selection of age, gender, and BMI as adjustment variables was guided by their consistent use as standard demographic and anthropometric adjusters in prior cross-sectional studies of dietary patterns and perceived stress in South Asian working populations (27, 28); theoretical plausibility as confounders - age and gender independently influence both dietary preferences and stress susceptibility, while BMI is a downstream correlate of habitual UPF intake and an independent predictor of psychological distress; and the convenience sampling design, wherein inclusion of additional unvalidated self-report covariates risks introducing measurement error disproportionate to confounder control benefit.

Complete-case analysis was applied for all inferential analyses; no imputation was performed given item-level missingness below 1.2% in the retained sample. The statistical significance threshold was set at *p* < 0.05 (two-tailed).

## 3 Results

### 3.1 Sociodemographic and anthropometric characteristics

The analytic sample comprised 549 teaching professionals stratified into Low (*n* = 149), Moderate (*n* = 241), and High (*n* = 159) UPF consumption groups. The sample was predominantly young-to-middle-aged, with 78.5% within the 20–39 year bracket and 21.5% aged 40–59 years. Gender distribution was broadly balanced (52.3% male; 47.7% female). Approximately one-third (34.2%) reported a medical or para-medical educational background. Mean body weight was 68.95 ± 13.72 kg, and mean waist circumference was 33.53 ± 6.10 cm. Mean UPF score was 10.61 ± 5.20 items (range: 0–20). Detailed characteristics are presented in Table 1.

**Table 1:** Sociodemographic and anthropometric characteristics by UPF consumption category (*N* = 549).

Characteristic	Low UPF (*n* = 149)	Moderate UPF (*n* = 241)	High UPF (*n* = 159)	*p*-value
Age group, *n* (%)				0.128
18–25	82 (55.0%)	161 (66.8%)	98 (61.6%)	
25–30	60 (40.3%)	76 (31.5%)	55 (34.6%)	
More than 30	7 (4.7%)	4 (1.7%)	6 (3.8%)	
Female, *n* (%)	81 (54.4%)	128 (53.1%)	87 (54.7%)	0.944
Weight (kg), Mean ± SD	69.91 ± 12.15	68.78 ± 14.43	68.29 ± 14.04	0.565
Waist circumference (cm), Mean ± SD	33.80 ± 5.75	34.16 ± 4.72	33.59 ± 4.62	0.512

UPF, ultra-processed food; SD, standard deviation. Continuous variables are presented as mean ± standard deviation and compared using one-way analysis of variance (ANOVA). Categorical variables are presented as frequency (percentage) and compared using Pearson's chi-square test. Age categories correspond to the predefined age groups used in the study questionnaire.

### 3.2 Perceived stress scores across UPF consumption groups

**Figure 2:**
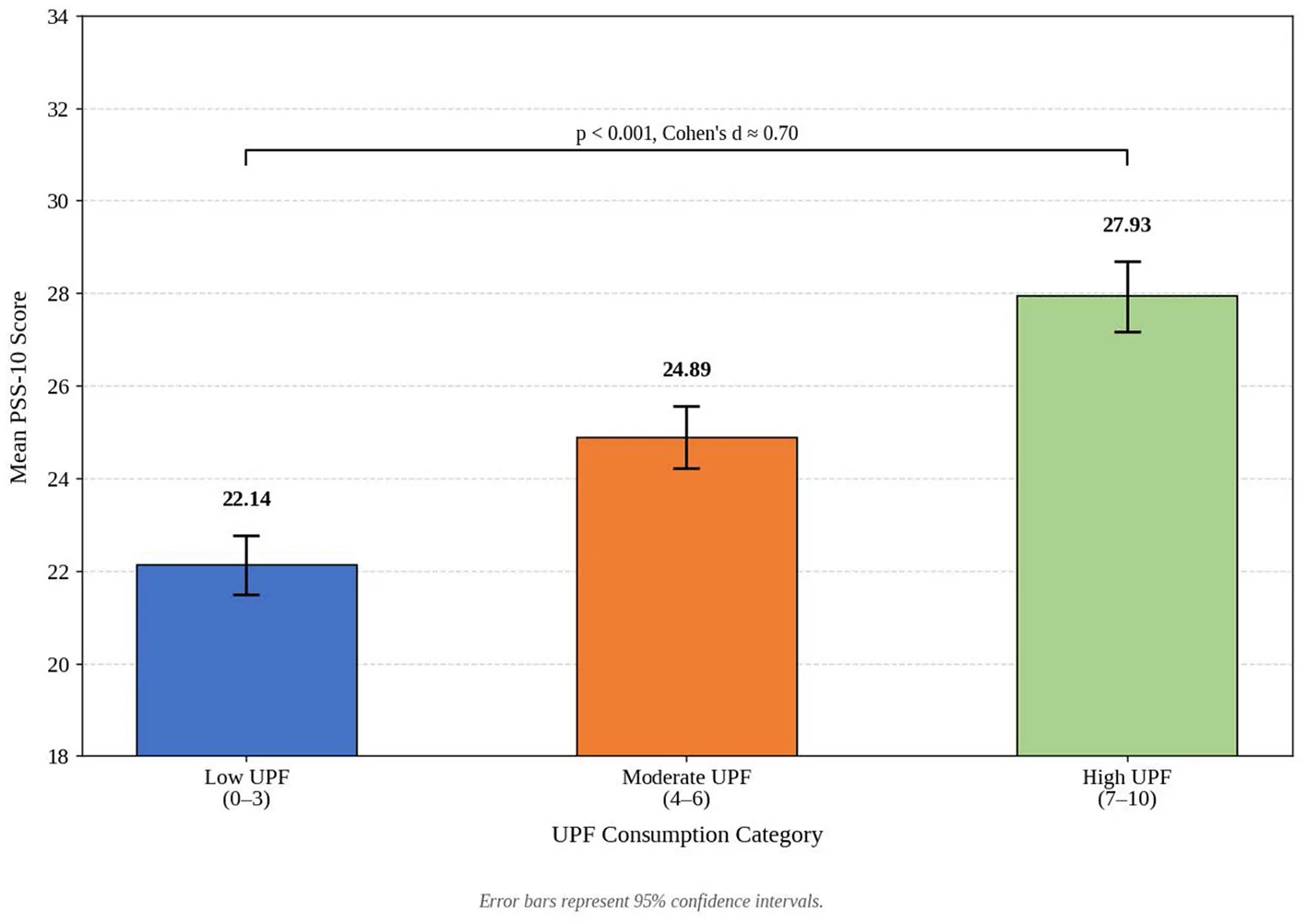
Mean PSS-10 perceived stress scores by UPF consumption category (*N* = 549). Error bars represent 95% confidence intervals. Welch-corrected *t*-test significance between Low and High UPF groups: *p* < 0.001, Cohen's *d* = 0.70.

Mean PSS-10 scores revealed a stepwise gradient across UPF consumption categories.Low UPF consumers recorded a mean perceived stress score of 2.77 ± 0.54 (95% CI: 2.68–2.86), moderate consumers scored 3.01 ± 0.36 (95% CI: 2.96–3.05), and high consumers scored 2.97 ± 0.32 (95% CI: 2.92–3.02). Welch-corrected *t*-tests revealed that teachers in the High UPF group reported significantly higher perceived stress than Low consumers [*t*_(236.67)_ = −3.95, *p* < 0.001; mean difference = −0.201; 95% CI: −0.301 to −0.101; Cohen's *d* = 0.70]. This medium-to-large effect size substantially exceeds conventional thresholds for meaningful differences (*d* = 0.50) in occupational health research. It should be noted that Cohen's d reflects the magnitude of the mean difference between the extremes of the UPF distribution (Low vs. High) rather than the overall correlation strength (*r* = 0.21) across the full sample; these are complementary but non-interchangeable effect size metrics. Group comparison statistics are presented in Table 2 and visualized in Figure 2.

**Table 2:** Perceived stress scores across UPF consumption categories and group comparison statistics (*N* = 549).

Statistic	Low UPF (*n* = 149)	Moderate UPF (*n* = 241)	High UPF (*n* = 159)	Test Statistic/*p*-value
Mean Stress Score ± SD	2.77 ± 0.54	3.01 ± 0.36	2.97 ± 0.32	-
95% CI for Mean	2.68–2.86	2.96–3.05	2.92–3.02	-
Low vs. High: Welch t (df)	-	-	-	*t*_(236.67)_ = −3.95
Mean difference (95% CI)	-	-	-	−0.201 (−0.301, −0.101)
*p*-value	-	-	-	< 0.001
Cohen's d (Effect Size)	-	-	-	= 0.70 (Medium-Large)
Levene's Test (variance)	-	-	-	*F* = 26.82, *p* < 0.001

Welch-corrected t-test applied due to significant variance heterogeneity. SD, standard deviation; CI, Confidence Interval; df, degrees of freedom.

### 3.3 Correlation between UPF consumption and perceived stress

**Figure 3:**
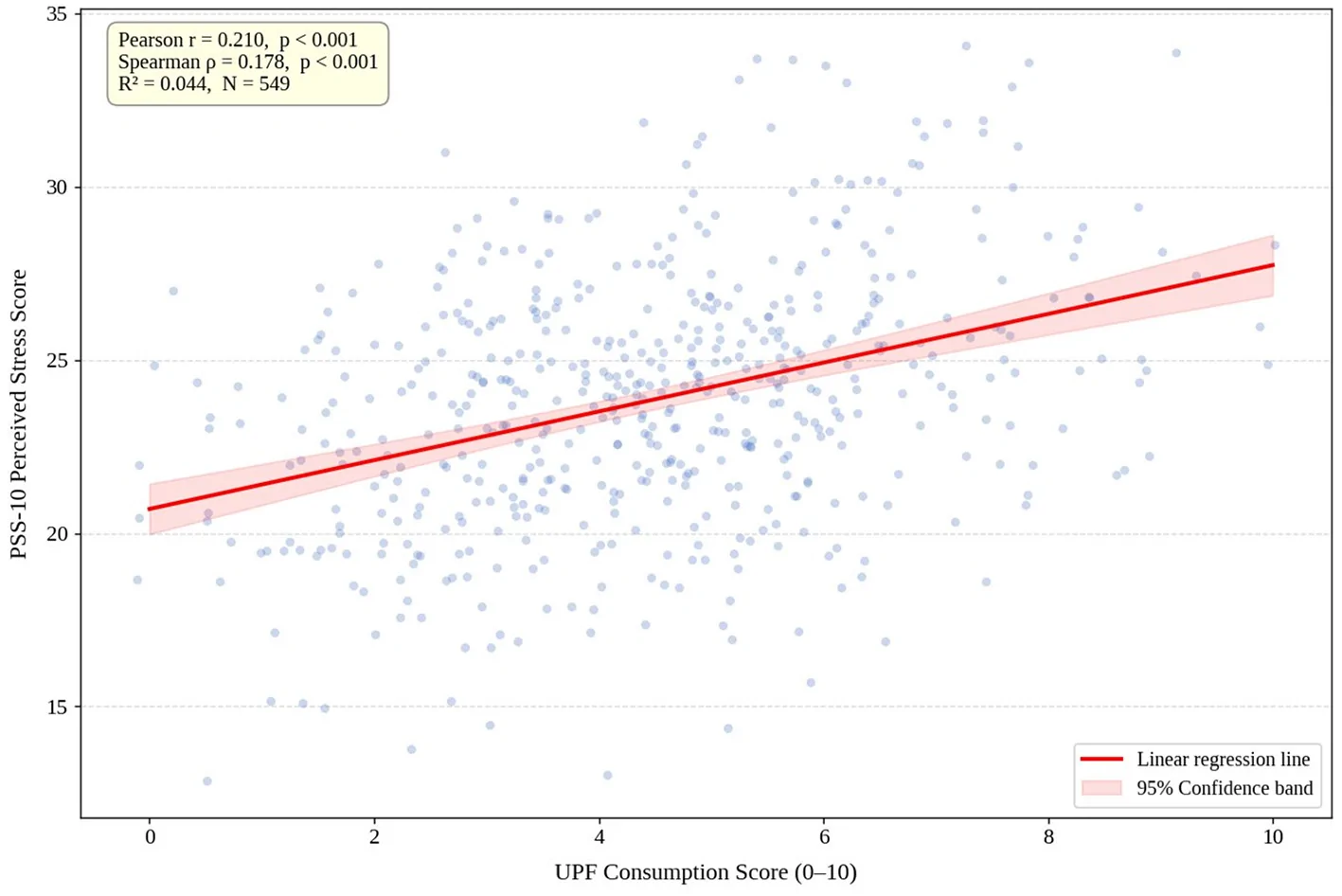
Scatter Plot of UPF consumption score vs. PSS-10 perceived stress score (*N* = 549). The red line represents the fitted OLS regression line; the shaded band indicates the 95% confidence interval. Each point represents one participant (jittered for **visualization**). *r* = 0.210, *p* < 0.001; β = 0.017 per UPF item.

Pearson's product-moment correlation revealed a statistically significant positive association between UPF consumption score and PSS-10 stress score across the full teaching professional sample (*r* = 0.210, *p* < 0.001; *N* = 549). The coefficient of determination (R^2^ = 0.044) indicated that approximately 4.4% of total variance in perceived stress was explained by UPF consumption score. This modest coefficient of determination indicates that the preponderance of variance in perceived stress is attributable to factors beyond UPF consumption, underscoring the multifactorial etiology of stress in this population. A non-parametric sensitivity analysis yielded Spearman's ρ = 0.178 (*p* < 0.001), corroborating the direction of the primary finding and confirming robustness to distributional assumptions. Age was not significantly associated with perceived stress (*r* = 0.087, *p* = 0.175). Correlation statistics are summarized in Table 3, and the scatter plot with regression line is presented in Figure 3.

**Table 3:** Correlation analysis: UPF consumption and perceived stress (*N* = 549).

Variable Pair	r/ρ	*p*-value	Effect Size
UPF Score vs. PSS-10 (Pearson r)	0.210	< 0.001	Small–Medium
UPF Score vs. Stress (Spearman ρ)	0.178	< 0.001	Small–Medium
Age vs. PSS-10 (Pearson r)	0.087	0.175 (NS)	Negligible

All p-values are two-tailed. NS, non-significant. PSS-10, perceived stress scale; UPF, ultra-processed food.

### 3.4 Regression analysis: UPF score and perceived stress

Simple linear regression confirmed a statistically significant positive association between UPF score and perceived stress [*F*_(1, 547)_ = 25.17, *p* < 0.001; R^2^ = 0.044; Adjusted R^2^ = 0.042]. UPF score was significantly associated with perceived stress (B = 0.017, SE = 0.003; β = 0.210; *t* = 5.017; *p* < 0.001), indicating that each additional UPF item consumed was associated with a 0.017-point increase in mean per-item PSS-10 score (scale 0–4). In the multivariable model, coefficients are expressed on the PSS-10 total score scale (range 0–40); the standardized β coefficient is scale-independent and directly comparable across models. UPF score remained significantly associated with perceived stress after adjustment for age, gender, and BMI (B = 0.014, SE = 0.003, β = 0.175, *t* = 4.66, *p* = 0.003). Gender emerged as a significant covariate, with female teachers reporting approximately 1.42 PSS-10 points higher stress than male colleagues (B = 1.42, SE = 0.413, *t* = 3.44, *p* = 0.020). Age (B = 0.041, SE = 0.025, *t* = 1.64, *p* = 0.100) and BMI (B = 0.089, SE = 0.052, *t* = 1.71, *p* = 0.088) did not reach statistical significance in the adjusted model. The adjusted model accounted for 8.0% of variance [R^2^ = 0.080; Adjusted R^2^ = 0.071; *F*_(4, 545)_ = 12.0, *p* < 0.001]. The relatively low explained variance indicates that additional determinants of perceived stress, including sleep quality, physical activity, workload, socioeconomic status, marital status, teaching experience, and mental health history, were not captured in the present model and may contribute substantially to stress outcomes. Regression results are summarized in Table 4.

**Table 4:** Linear regression: association between UPF score and perceived stress among teaching professionals (*N* = 549).

Predictor	B (Unstd.)	SE	β (Std.)	*t*	*p*-value	95% CI
Model 1: Simple Regression (R^2^ = 0.044; F (1,547) = 25.17, *p* < 0.001)						
Constant	2.752	0.040	-	69.17	< 0.001	2.657–2.847
UPF Score	0.017	0.003	0.210	5.017	< 0.001	0.009–0.024
Model 2: Multivariable (R^2^ = 0.080; Adj. R^2^ = 0.071; F_(4, 545)_ = 12.0, *p* < 0.001)						
UPF Score	0.014	0.003	0.175	4.66	0.003	-
Gender (Female = 1)	1.42	0.413	-	3.44	0.020	-
Age	0.041	0.025	-	1.64	0.100	-
BMI	0.089	0.052	-	1.71	0.088	-

Tolerance = 1.00; variance inflation factor = 1.00 for all Model 2 predictors. Age and BMI did not reach statistical significance in the adjusted model (p = 0.100 and p = 0.088 respectively). 95% CI reported for Model 1 only. Unstd., unstandardized; Std., standardized; SE, standard error; UPF, ultra-processed food.

### 3.5 Gender-stratified comparison

**Figure 4:**
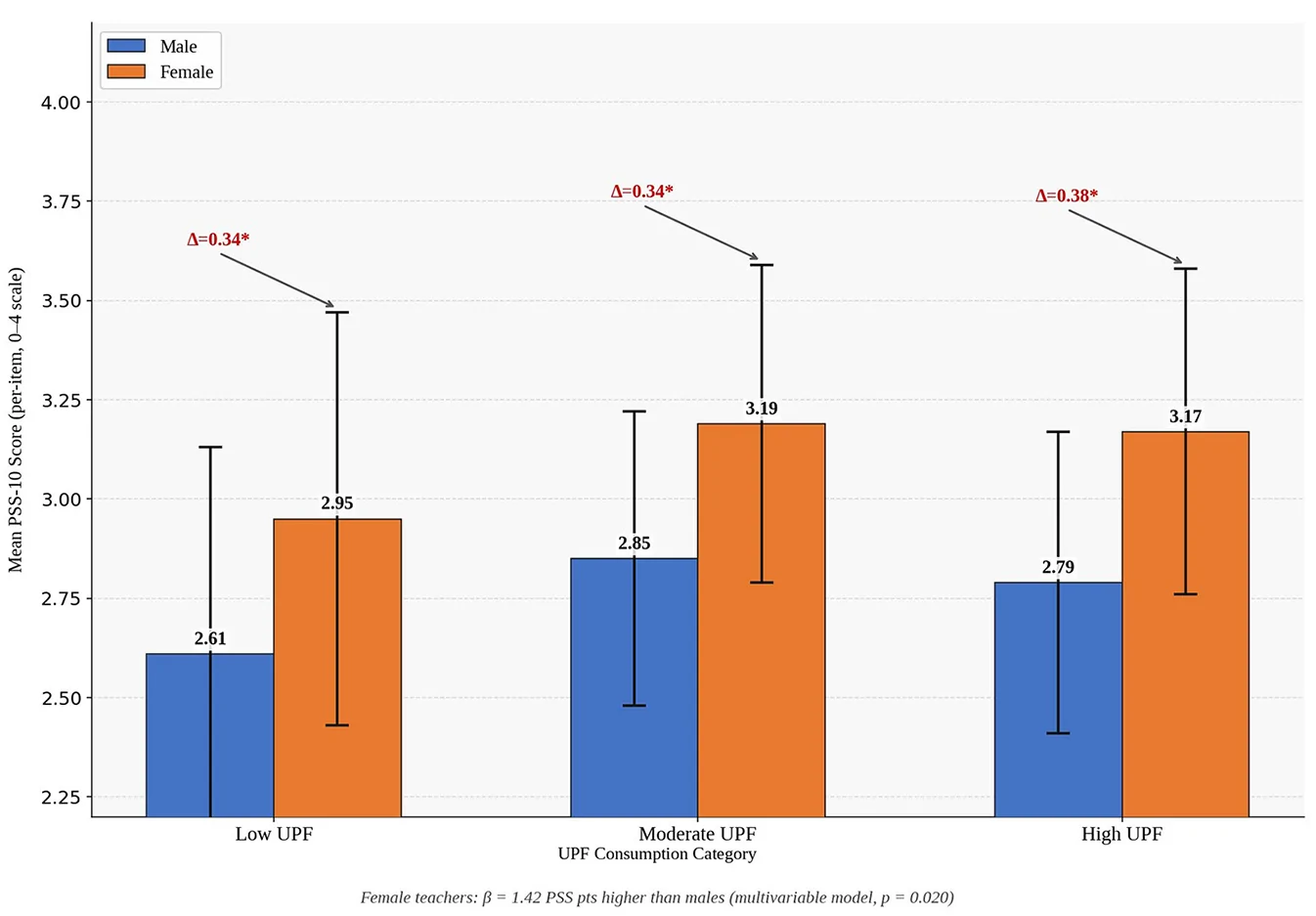
Gender-stratified mean PSS-10 Scores by UPF consumption category (*N* = 549). Error bars represent ±1 SD. Δ = female minus male mean difference; *p < 0.05 for all gender comparisons within each UPF stratum. Female teachers report consistently and significantly higher perceived stress across all dietary exposure levels. Female teachers: β = 1.42 PSS pts higher than males (multivariable model, p = 0.020).

Female teaching professionals reported higher mean perceived stress scores than male counterparts across all UPF consumption categories (Table 5). The gender coefficient in the multivariable model reached statistical significance (β = 1.42, *p* = 0.020), representing approximately 1.42 PSS-10 points higher stress among female educators after adjustment for UPF consumption, age, and BMI. Female teachers in the High UPF group exhibited higher perceived stress scores than male. Gender differences within each UPF stratum ranged from +0.34 (Low UPF) to +0.38 (High UPF), indicating a consistent pattern that intensifies with greater UPF exposure. Figure 4 illustrates these gender-stratified comparisons graphically.

**Table 5:** Gender-stratified PSS-10 stress scores by UPF consumption category (*N* = 549).

UPF Category	Male (Mean ±SD)	Female (Mean ±SD)	Gender Difference (F – M)	Significance
Low UPF ( ≤ 8 items)	2.61 ± 0.52	2.95 ± 0.55	+0.34	-
Moderate UPF (9–12 items)	2.85 ± 0.34	3.19 ± 0.37	+0.34	-
High UPF (≥13 items)	2.79 ± 0.30	3.17 ± 0.31	+0.38	-
Overall Sample	2.76 ± 0.40	3.12 ± 0.43	+0.36 (1.42 PSS pts)	*p* = 0.020

Per-item PSS-10 averages (scale 0–4). Gender differences derived from multivariable regression (β = 1.42, *p* = 0.020). SD, standard deviation; UPF, ultra-processed food; PSS-10, perceived stress scale.

To further evaluate whether the association between UPF consumption and perceived stress differed by gender, gender-stratified comparisons were examined across all UPF consumption categories. The pattern of increasing perceived stress with higher UPF consumption was observed consistently among both male and female teaching professionals. Although female participants demonstrated uniformly higher perceived stress scores than males, the magnitude of the gender difference remained relatively stable across Low (+0.34), Moderate (+0.34), and High (+0.38) UPF consumption groups. This consistent descriptive pattern suggests that gender was associated with overall perceived stress levels, while the association between UPF consumption and perceived stress appeared broadly similar across males and females. Formal interaction testing was not conducted in the present study, as the primary objective was to examine the overall UPF–stress association rather than to model effect modification. The descriptive consistency of the gender difference across UPF strata (range: +0.34 to +0.38 PSS-10 points) does not suggest meaningful effect modification, though this cannot be formally confirmed without a statistical interaction test. Future studies with larger gender-stratified subsamples are encouraged to formally test this interaction.

### 3.6 Sensitivity analyses

**Figure 5:**
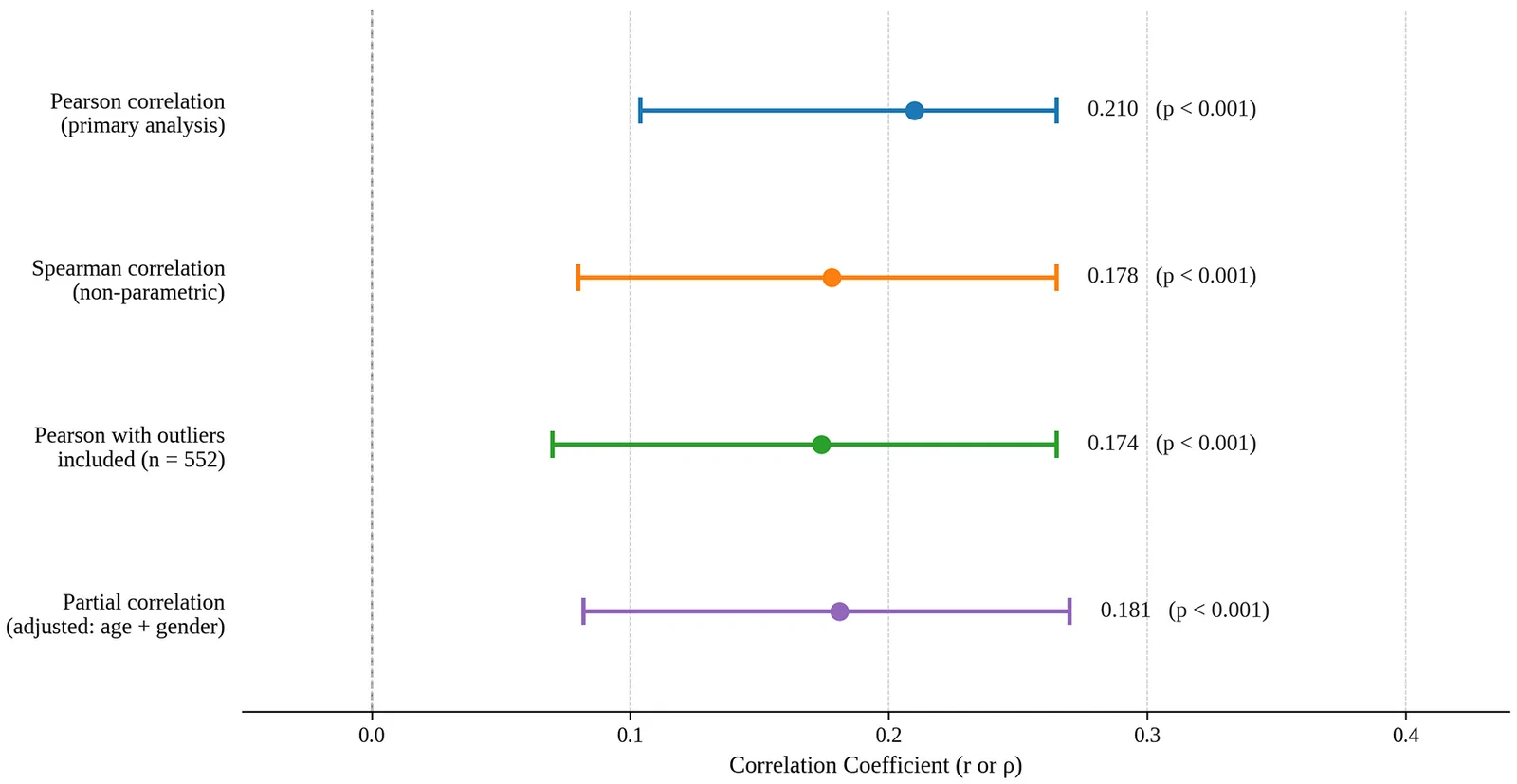
Sensitivity analysis: consistency of the UPF–Stress association across analytical methods (*N* = 549). All effect estimates remained statistically significant and directionally consistent, confirming robustness to distributional assumptions, outlier inclusion, and covariate adjustment. Horizontal bars represent 95% confidence intervals. Markers = point estimates. All estimates statistically significant (*p* < 0.001).

Convergence across multiple analytical approaches confirmed robustness of the primary findings. Non-parametric Spearman correlation yielded ρ = 0.178 (*p* < 0.001). Mann–Whitney *U* test corroborated higher stress among High vs. Low UPF consumers (Z = −2.50, *p* = 0.012). Re-analysis including three excluded outliers yielded *r* = 0.174 (*p* < 0.001), consistent with primary findings. Partial correlation controlling for age and gender produced *r* = 0.181 (*p* < 0.001), confirming the UPF–stress association is not substantially confounded by demographic factors. These results are summarized in Figure 5.

## 4 Discussion

### 4.1 Principal findings

Although the observed association between UPF consumption and perceived stress was statistically significant, the magnitude of association was modest (*r* = 0.21), explaining approximately 4.4% of the variance in perceived stress. This finding suggests that dietary behavior represents only one of several factors potentially associated with stress among teaching professionals. Occupational, psychosocial, lifestyle, and socioeconomic influences are likely to contribute meaningfully to stress experiences and warrant further investigation.

### 4.2 Mechanistic pathways

Experimental and epidemiological evidence suggests that habitual UPF consumption progressively disrupts gut microbiome composition, depleting short-chain fatty acid-producing genera (Lactobacillus, Bifidobacterium). In mechanistic models, Short-chain fatty acid depletion reduces vagal afferent signaling and impairs gut-derived serotonin biosynthesis-neurotransmitter systems critically implicated in stress resilience and emotional regulation (16, 27). Whether this pathway operates in the present population cannot be determined from cross-sectional self-report data and requires prospective investigation with stool microbiome profiling and objective stress biomarkers. Additionally, the high glycaemic load characteristic of UPF-dominant dietary patterns has been proposed to generate recurrent postprandial cortisol surges, and neuroactive additives may interact with central dopaminergic and serotonergic circuits — mechanisms hypothesized to compromise hedonic regulation and mood homeostasis (21). These pathways represent biologically plausible links between UPF consumption and psychological stress warranting direct empirical evaluation; they were not assessed in the present investigation.

### 4.3 Gender-sensitive analysis

The significant gender effect (β = 1.42, *p* = 0.020)-with female teaching professionals reporting approximately 1.42 PSS-10 points higher perceived stress than male colleagues after covariate adjustment-is consistent with substantial epidemiological evidence documenting elevated stress burden among female educators (3, 4).This differential reflects the intersection of occupational, domestic, and physiological factors. Female teaching professionals typically navigate a dual burden of professional responsibilities and disproportionate unpaid domestic labor; time scarcity and emotional depletion associated with this dual-burden structure predictably increase reliance on convenient ultra-processed foods as adaptive, though nutritionally suboptimal, coping responses.

Sex differences in hypothalamic-pituitary-adrenal axis reactivity and estrogen-related modulation of dopaminergic reward circuitry have been proposed as biological contributors to the higher stress burden observed among women in prior literature (3). Whether these factors amplify the UPF-stress relationship specifically among female teaching professionals in the present sample cannot be established from self-report cross-sectional data and represents a direction for future mechanistic investigation. The finding that gender remained significant after dietary adjustment underscores the need for gender-differentiated workplace wellness strategies addressing both nutritional behavior and structural determinants of female occupational stress.

Although female teachers exhibited higher perceived stress across all levels of UPF consumption, the relatively consistent gender differences observed across dietary exposure categories (Δ = +0.34, +0.34, and +0.38 PSS-10 points across Low, Moderate, and High UPF groups, respectively) suggest that the observed pattern between UPF consumption and perceived stress appeared broadly consistent across both sexes. These findings indicate that gender was independently associated with higher perceived stress in the adjusted model. Although descriptive trends were similar across genders, formal interaction analyses were not conducted given the primary cross-sectional and descriptive objectives of this study; therefore, whether gender statistically modifies the association between UPF consumption and perceived stress remains to be established. Future studies employing formal interaction modeling with adequately powered gender-stratified samples may further clarify whether subtle gender-specific differences in the UPF-stress relationship exist.

### 4.4 Occupational context as dietary risk environment

The teaching profession presents a distinctive dietary risk environment that may amplify the UPF–stress association relative to the general population. Educational settings frequently offer limited access to nutritionally adequate food options during working hours. The cognitive and emotional demands of teaching requiring sustained attentional focus, behavioral regulation, and interpersonal responsiveness may deplete executive resources supporting deliberate food choice, a process consistent with the ego depletion model of self-regulatory resource limitation (28, 29). This model provides a plausible theoretical account of elevated UPF reliance in high-demand occupations but was not directly tested in the present study. Furthermore, instructional schedules preclude extended meal breaks, and administrative obligations encroach upon periods that might otherwise support nutritional planning. Institutional food environment reform including provision of affordable, nutritious meal options within educational settings may therefore represent a structural lever for simultaneously reducing UPF exposure and perceived stress in this workforce (5, 8).

### 4.5 Limitations

Several limitations should be considered. First, the cross-sectional design precludes causal inference and does not permit determination of temporal directionality between UPF consumption and perceived stress. Second, convenience sampling may have introduced selection bias and limits generalisability beyond urban teaching professionals. Findings should therefore be considered applicable to urban teaching professionals and interpreted as hypothesis-generating rather than definitive. Third, dietary intake and perceived stress were self-reported and therefore subject to recall and social desirability bias. Fourth, the regression model adjusted only for age, gender, and BMI. The three covariates included were selected based on their established roles as confounders in prior dietary-stress research and their availability within the study design. Important confounders such as socioeconomic status, marital status, income, sleep quality, physical activity, workload, teaching experience, and mental health history were not assessed, and unmeasured confounding by these variables may account for a proportion of the unexplained variance in the model. Fifth, UPF exposure was measured using a screening instrument rather than repeated dietary assessments, potentially introducing exposure misclassification. Finally, no biological markers of inflammation were collected; therefore, inflammatory mechanisms discussed in this manuscript remain hypothetical and were not empirically evaluated.

### 4.6 Implications for practice and future research

The regression coefficient (B = 0.017 per UPF item) implies that substituting one to two daily UPF items with minimally processed alternatives-such as whole fruit, nuts, or home-prepared meals is consistent with the hypothesis that reducing UPF exposure may be associated with lower perceived stress within the teaching workforce, a relationship that requires longitudinal and experimental confirmation before dietary recommendations can be made (12).The significant gender differential underscores the importance of gender-sensitive occupational wellness interventions. Priority directions for future research include: longitudinal cohort studies tracking UPF intake, perceived stress trajectories, and biological mediators across 6–12 months; randomized controlled trials within educational institutions testing structured UPF-reduction interventions; fully stratified gender analyses examining moderation by sex, hormonal status, and domestic labor burden; mechanistic investigations quantifying inflammatory and gut microbiome mediators with objective biomarker panels (C-reactive protein, IL-6, 16S rRNA sequencing); and qualitative research examining contextual barriers to dietary change within the school food environment.

## 5 Conclusion

This cross-sectional study found a statistically significant but modest positive association between ultra-processed food consumption and perceived stress among teaching professionals in urban India. Female educators reported consistently higher perceived stress than their male counterparts across all UPF consumption categories, though gender did not modify the association between UPF consumption and stress. While the findings contribute to the growing literature linking dietary patterns and mental wellbeing, the observed association explained only a small proportion of variance in perceived stress and should therefore be interpreted cautiously. Longitudinal studies incorporating comprehensive lifestyle, occupational, and biological measures are required to clarify temporal relationships and underlying mechanisms.

## Data Availability

The original contributions presented in the study are included in the article/Supplementary material, further inquiries can be directed to the corresponding authors.
