# Supplementary material for: Association between ultra-processed food consumption and perceived stress among teaching professionals: a gender-stratified cross-sectional study from Urban India
Source: Front Nutr. 2026 Aug 21;13:1889456. doi: 10.3389/fnut.2026.1889456 (PMC13541530; doi:10.3389/fnut.2026.1889456)
Supplement: Supplementary file 1 [file Supplementary_File_1.pdf]

Association Between Ultra-Processed Food Consumption and Perceived Stress Among Teaching Professionals: A Gender-Stratified Cross-Sectional Study from Urban India

STROBE Checklist

Section/Topic	Item	Recommendation	Section Reference	Relevant Text from Manuscript
**TITLE AND ABSTRACT**				
**Title & Abstract**	1a	Indicate the study's design with a commonly used term in the title or the abstract	Title; Abstract (Methods)	Title: 'Association Between Ultra-Processed Food Consumption and Perceived Stress Among Teaching Professionals: A Gender-Stratified Cross-Sectional Study from Urban India.' The term 'cross-sectional study' appears explicitly in the title. The Abstract Methods section also states: 'A cross-sectional analytical study was conducted among 549 teaching professionals.'
	1b	Provide in the abstract an informative and balanced summary of what was done and what was found	Abstract	The Abstract contains structured Background, Objective, Methods, Results, and Conclusion sections. Methods: study design, sample (N=549), UPF instrument (20-item NOVA-based checklist), outcome (PSS-10), and analytical methods stated. Results: group-wise PSS-10 means, Welch t-test (t(236.67)=−3.95, p<0.001, d≈0.70), Pearson r=0.210 (p<0.001, R^2=0.044), Spearman ρ=0.178, multivariable β=0.175 (p=0.003), gender effect (B=1.42, SE=0.413, t=3.44, p=0.020). Formal interaction testing was not conducted; gender-stratified descriptive comparisons showed stable gender differences across UPF strata (+0.34 to +0.38 PSS-10 points). Conclusion: statistically significant but modest association; mechanisms not empirically tested; longitudinal biomarker-inclusive research warranted.
**INTRODUCTION**				
**Background / Rationale**	2	Explain the scientific background and rationale for the investigation being reported	Introduction (Section 1)	Section 1 establishes: (1) rising UPF consumption in urban India and globally; (2) high occupational stress burden among teaching professionals (prevalence 8.3–87.1% across contexts); (3) gendered stress vulnerability; (4) proposed biological pathways linking UPFs to psychological stress (HPA axis activation, gut-brain axis disruption, neuroactive additives, glycaemic load); (5) gap: no prior study examines UPF–stress association specifically in Indian teaching professionals. References 1–22 and 32 cited throughout.
**Objectives**	3	State specific objectives, including any prespecified hypotheses	Introduction (final paragraph); Sections 3.5, 4.3	Introduction final paragraph: 'The present study was designed to examine the association between ultra-processed food consumption and perceived stress levels among teaching professionals, employing validated instruments within a cross-sectional analytical framework.' Secondary objective: to evaluate gender-related differences in dietary behaviour and stress experience through gender-stratified descriptive comparisons, as stated in Sections 3.5 and 4.3. Hypothesis: higher UPF consumption is positively associated with perceived stress, with gender as an independent predictor of stress burden.
**METHODS**				
**Study Design**	4	Present key elements of study design early in the paper	Section 2.1	Section 2.1: 'A cross-sectional analytical study was conducted between September and December 2025 across selected educational institutions and community centres in Pune, Maharashtra, India. This design enables simultaneous assessment of exposure (UPF consumption) and outcome (perceived stress) variables within a single timeframe, supporting hypothesis generation for future longitudinal investigation.'
**Setting**	5	Describe the setting, locations, and relevant dates, including periods of	Sections 2.1, 2.3	Section 2.1: Setting: educational institutions and community centres in Pune, Maharashtra, India. Period: September–December 2025. Section 2.3: Questionnaires distributed to 631 teaching professionals across

Section/Topic	Item	Recommendation	Section Reference	Relevant Text from Manuscript
		recruitment, exposure, follow-up, and data collection		14 educational institutions in Pune. Cross-sectional design: exposure and outcome measured simultaneously at a single time point; no follow-up period applicable.
**Participants**	6a	Cross-sectional study — Give the eligibility criteria, and the sources and methods of selection of participants	Section 2.3	Section 2.3: Convenience sampling employed across 14 educational institutions in Pune. Inclusion criteria: (1) currently employed as a teaching professional; (2) aged 18–60 years; (3) capable of independently completing structured questionnaires; (4) provision of voluntary written informed consent. Exclusion criteria: (1) current diagnosis of an eating disorder; (2) severe mental health condition precluding reliable self-report; (3) inability to provide informed consent; (4) incomplete questionnaire responses (>20% missing items). Recruitment flow: 631 approached → 82 excluded (34 incomplete questionnaires; 26 ineligible — 18 non-teaching administrative roles, 8 outside age range; 22 declined) → 549 final analytic sample (response rate: 87.0%).
	6b	Not applicable — cross-sectional study (matching not used)	Not applicable	Not applicable. This is a cross-sectional study. No matched design was employed. All 549 eligible participants were included in the analytic sample without matching.
**Variables**	7	Clearly define all outcomes, exposures, predictors, potential confounders, and effect modifiers. Give diagnostic criteria, if applicable	Sections 2.4, 2.5	PRIMARY EXPOSURE — UPF Consumption Score: 20-item researcher-developed binary checklist derived from NOVA Group 4 food categories, adapted to the Indian urban dietary context. Each item coded 1 (consumed) or 0 (not consumed). Total score range: 0–20. Categorised as Low (≤8), Moderate (9–12), and High (≥13). PRIMARY OUTCOME — Perceived Stress: 10-item Perceived Stress Scale (PSS-10). Five-point Likert scale (0=Never to 4=Very Often). Items 4, 5, 7, 8 reverse-scored. Total score 0–40; mean per-item score (0–4) used in Model 1 analyses. Higher scores = greater perceived stress. COVARIATES/CONFOUNDERS: Age (continuous, years), Gender (binary: male=0, female=1), BMI (continuous, kg/m²). Selected a priori based on established roles as confounders in dietary-stress research. GENDER AS COVARIATE: Gender was examined as a potential independent predictor via the adjusted regression model (Model 2). Formal interaction testing was not conducted; gender-stratified descriptive comparisons are presented in Table 6 and Section 3.5. ADDITIONAL DESCRIPTORS: Weight (kg), waist circumference (cm), health literacy background (medical/para-medical vs. other), collected for sample characterisation.
**Data Sources / Measurement**	8	For each variable of interest, give sources of data and details of methods of assessment (measurement). Describe comparability of assessment methods if there is more than one group	Sections 2.4, 2.5	UPF EXPOSURE (Section 2.4): Researcher-developed 20-item checklist. Content validity established by panel of three registered nutrition professionals; piloted in 30 urban teaching professionals. Self-administered questionnaire; binary response per item. 24-hour dietary recall collected for descriptive dietary characterisation only — not used in inferential analyses. PERCEIVED STRESS (Section 2.5): PSS-10 (Cohen, Kamarck & Mermelstein, 1983). Self-administered. Cronbach's α=0.78–0.91; test-retest reliability r>0.70; validated in South Asian populations (Rajaa et al., 2022). Reverse-scoring applied to items 4, 5, 7, 8 per standard protocol. ANTHROPOMETRIC DATA: Self-reported body weight and waist circumference recorded on the structured questionnaire. BMI computed from self-reported weight and height. All instruments administered in English. Assessment methods were identical across all participants; no group-specific measurement differences applied.
**Bias**	9	Describe any efforts to address potential sources of bias	Sections 2.3, 2.6, 4.5	SELECTION BIAS: Convenience sampling acknowledged as a limitation. Response rate of 87.0% (549/631) minimises non-response bias. Demographic comparison with National Sample Survey (2022–23) confirms broad consistency with urban private-sector teacher profile. INFORMATION BIAS: Both exposure and outcome are self-reported, introducing potential recall and social desirability bias. The PSS-10 is a well-validated instrument with established reliability in South Asian populations, reducing outcome measurement error. The UPF checklist was content-validated and piloted prior to use. CONFOUNDING: Multivariable regression adjusting for age, gender, and BMI applied. Residual confounding by unmeasured

Section/Topic	Item	Recommendation	Section Reference	Relevant Text from Manuscript
				variables (SES, sleep quality, physical activity, workload, mental health history) acknowledged in Section 4.5. MISSING DATA BIAS: Participants with >20% missing items excluded (n=34). Item-level missingness in retained sample <1.2% of cells across all variables. Complete-case analysis applied; no imputation performed.
**Study Size**	10	Explain how the study size was arrived at	Section 2.3	Section 2.3: The target sample size was pre-specified to achieve 80% statistical power for detection of a moderate effect size (r ≈ 0.15) at a two-tailed significance threshold of α = 0.05, requiring a minimum of 348 participants (computed using G*Power for bivariate correlation). The achieved sample of 549 provided greater than 95% power for the primary correlation analysis. Power calculation based on Cohen (1988) conventions for medium effect in correlation analysis.
**Quantitative Variables**	11	Explain how quantitative variables were handled in the analyses. If applicable, describe which groupings were chosen and why	Sections 2.4, 3.1, 3.7	UPF SCORE (continuous, 0–20): Used as continuous variable in all regression and correlation analyses. Also categorised into three groups: Low (≤8 items), Moderate (9–12 items), High (≥13 items). Category boundaries based on approximate tertile distribution facilitating clinical interpretability. PSS-10 SCORE: Used as continuous outcome. Mean per-item score (0–4) used in Model 1; total score (0–40) used in Model 2. Group means presented as mean ± SD with 95% CI. AGE: Continuous variable in regression models. Presented in age-group categories (18–25, 25–30, >30) in Table 1 for descriptive purposes. BMI: Continuous variable in regression models. Outliers: Three identified using IQR criterion; excluded from primary analysis. Sensitivity analysis including outliers yielded r=0.174 (p<0.001), consistent with primary finding (Section 3.7).
**Statistical Methods**	12a	Describe all statistical methods, including those used to control for confounding	Section 2.6	All analyses performed in R (version 4.3.1). Normality: Shapiro–Wilk test and Q–Q plots. Group comparisons: Welch-corrected independent samples t-test applied due to confirmed variance heterogeneity (Levene's test F=26.82, p<0.001). Correlation: Pearson's product-moment correlation (primary); Spearman's ρ (non-parametric sensitivity check). Simple linear regression (Model 1): UPF Score as sole predictor of PSS-10 mean per-item score (scale 0–4). Multivariable linear regression (Model 2): UPF Score adjusted for age, gender, and BMI — outcome expressed on PSS-10 total score scale (0–40). Standardised β is scale-independent. Statistical significance threshold: p<0.05, two-tailed throughout.
	12b	Describe any methods used to examine subgroups and interactions	Sections 3.5, 4.3	Sections 3.5 and 4.3: Gender-stratified descriptive comparisons of PSS-10 scores across all UPF consumption categories reported in Table 6. Formal interaction modelling was not conducted, as the primary objective was to examine the overall UPF–stress association. The descriptive stability of gender differences across UPF strata (range: +0.34 to +0.38 PSS-10 points) does not suggest meaningful effect modification, though this cannot be formally confirmed without statistical interaction testing. Future studies are encouraged to formally test this interaction.
	12c	Explain how missing data were addressed	Sections 2.3, 2.6	Section 2.3 and 2.6: Participants with >20% missing questionnaire items were excluded prior to analysis (n=34). Among the 549 retained participants, item-level missingness was <1.2% of cells across all variables. Complete-case analysis applied for all inferential analyses; no imputation performed given the very low rate of item-level missingness.
	12d	Cross-sectional study — If applicable, describe analytical methods taking account of sampling strategy	Sections 2.3, 4.5	Convenience sampling employed. No complex survey design (clustering, stratification, or probability weights) was used; therefore no design-based variance estimation was required. All standard errors reflect simple random sampling assumptions. Limitation of convenience sampling and its potential impact on generalisability are explicitly discussed in Section 4.5. The achieved demographic profile was compared with National Sample Survey data to contextualise the sample composition.

Section/Topic	Item	Recommendation	Section Reference	Relevant Text from Manuscript
	12e	Describe any sensitivity analyses	Section 3.7; Figure 5	Section 3.7: (1) Non-parametric Spearman correlation: ρ=0.178, p<0.001 — consistent with Pearson r=0.210. (2) Mann–Whitney U test: Z=−2.50, p=0.012 — corroborated higher stress in High vs Low UPF group. (3) Outlier sensitivity: re-analysis including 3 excluded outliers yielded r=0.174, p<0.001 — consistent. (4) Partial correlation controlling for age and gender: r=0.181, p<0.001 — UPF–stress association not substantially confounded by demographics. These results are summarised in Figure 5 (Sensitivity Analysis figure).
**RESULTS**				
**Participants**	13a	Report numbers of individuals at each stage of study	Section 2.3; Table 1	Approached: 631 teaching professionals across 14 educational institutions. Excluded: 82 total — 34 incomplete questionnaires (>20% missing); 26 ineligible (18 non-teaching administrative roles; 8 outside age range 18–60 years); 22 declined participation after receiving study information. Final analytic sample: 549 participants (response rate: 87.0%). UPF consumption groups: Low (n=149), Moderate (n=241), High (n=159). Three outliers excluded from primary analysis; included in sensitivity analysis (Section 3.7).
	13b	Give reasons for non-participation at each stage	Section 2.3	Section 2.3: Three reasons for non-participation: (1) Incomplete questionnaires — >20% missing items (n=34); (2) Ineligibility — non-teaching administrative roles (n=18) or age outside specified range (n=8); (3) Voluntary declination after receiving study information (n=22). Total excluded: n=82.
	13c	Consider use of a flow diagram	Supplementary Figure 1	A participant flow diagram is provided as Supplementary Figure 1. The numerical flow is described in Section 2.3 (631 approached → 82 excluded → 549 analysed).
**Descriptive Data**	14a	Give characteristics of study participants and information on exposures and potential confounders	Table 1	Table 1 presents sociodemographic and anthropometric characteristics by UPF consumption category (N=549): Age group (p=0.128): 18–25 — Low 55.0%, Moderate 66.8%, High 61.6%; 25–30 — Low 40.3%, Moderate 31.5%, High 34.6%; >30 — Low 4.7%, Moderate 1.7%, High 3.8%. Gender female % — Low 54.4%, Moderate 53.1%, High 54.7% (p=0.944). Weight: Low 69.91±12.15, Moderate 68.78±14.43, High 68.29±14.04 kg (p=0.565). Waist circumference: Low 33.80±5.75, Moderate 34.16±4.72, High 33.59±4.62 cm (p=0.512). Mean UPF score: 10.61±5.20 (range 0–20). Mean PSS-10 per-item: 2.77±0.54 (Low), 3.01±0.36 (Moderate), 2.97±0.32 (High).
	14b	Indicate number of participants with missing data for each variable of interest	Sections 2.3, 2.6	Section 2.3 and 2.6: Item-level missingness among 549 retained participants was minimal: <1.2% of cells across all variables. PSS-10 had 7 partially missing item responses; UPF checklist had 11 partially missing item responses. All retained participants had complete data for the primary exposure (UPF score) and primary outcome (PSS-10 total). No imputation was performed.
	14c	Not applicable — cross-sectional study (no follow-up)	Not applicable	Not applicable. This is a cross-sectional study. All data were collected at a single time point (September–December 2025). There was no follow-up period.
**Outcome Data**	15	Cross-sectional study — Report numbers of outcome events or summary measures	Table 2; Section 3.2	Table 2 and Section 3.2: Perceived stress scores (PSS-10 mean per-item, scale 0–4) by group — Low UPF: 2.77±0.54 (95% CI: 2.68–2.86); Moderate UPF: 3.01±0.36 (95% CI: 2.96–3.05); High UPF: 2.97±0.32 (95% CI: 2.92–3.02). Overall sample: 78.5% aged 20–39 years; 52.3% male, 47.7% female. Welch t-test (High vs Low): t(236.67)=−3.95, p<0.001, mean difference=−0.201 (95% CI: −0.301 to −0.101), Cohen's d≈0.70.
**Main Results**	16a	Give unadjusted and confounder-adjusted estimates and their precision. Make clear which confounders were adjusted for and why	Table 4; Section 3.3	UNADJUSTED (Model 1, Table 4, per-item scale 0–4): B=0.017, SE=0.003, β=0.210, t=5.017, p<0.001; 95% CI: 0.009–0.024; R^2=0.044, F(1,547)=25.17, p<0.001. ADJUSTED (Model 2, Table 4, total score scale 0–40): UPF Score — B=0.175, SE=0.059, β=0.175, t=2.97, p=0.003. Gender (Female=1) — B=1.42, SE=0.413, t=3.44, p=0.020. Age — B=0.041, SE=0.025, t=1.64, p=0.100 (NS). BMI — B=0.089, SE=0.052, t=1.71, p=0.088 (NS). R^2=0.080; Adj. R^2=0.071; F(4,545)=12.0, p<0.001. NOTE: Model 1 and Model 2 use different

Section/Topic	Item	Recommendation	Section Reference	Relevant Text from Manuscript
				PSS-10 scales (per-item vs total). Standardised β is scale-independent and directly comparable across models. CONFOUNDERS: Age — independently influences dietary preferences and stress susceptibility; Gender — well-established determinant of stress; BMI — downstream correlate of habitual UPF intake. Selection guided by prior literature in South Asian working populations (refs 28, 29). CORRELATION: Pearson r=0.210 ($p<0.001$; N=549); Spearman ρ=0.178 ($p<0.001$).
	16b	Report category boundaries when continuous variables were categorised	Sections 2.4, 3.1	Section 2.4 and 3.1: UPF consumption score (0–20) categorised as: Low ≤8 items (n=149), Moderate 9–12 items (n=241), High ≥13 items (n=159). Boundaries based on approximate tertile distribution. Age categorised descriptively as: 18–25, 25–30, and >30 years in Table 1, corresponding to predefined questionnaire age groups.
	16c	If relevant, consider translating estimates of relative risk into absolute risk	Not applicable	Not applicable. The outcome (perceived stress, PSS-10 score) is a continuous variable analysed using linear regression. Absolute differences in PSS-10 scores between groups are reported directly: mean difference Low vs High UPF = −0.201 PSS-10 per-item points; gender effect = +1.42 PSS-10 total score points for female vs male educators.
**Other Analyses**	17	Report other analyses done — eg subgroups, interactions, sensitivity analyses	Section 3.5, Table 6; Section 3.7	GENDER SUBGROUP ANALYSIS (Section 3.5, Table 6): PSS-10 scores stratified by gender across all UPF categories. Female: Low 2.95±0.55, Moderate 3.19±0.37, High 3.17±0.31. Male: Low 2.61±0.52, Moderate 2.85±0.34, High 2.79±0.30. Gender difference stable: +0.34 to +0.38 PSS-10 points across strata. Formal interaction analysis not conducted; descriptive pattern does not suggest meaningful effect modification. SENSITIVITY ANALYSES (Section 3.7): Spearman ρ=0.178 ($p<0.001$); Mann–Whitney Z=−2.50 (p=0.012); outlier-inclusive r=0.174 ($p<0.001$); partial correlation r=0.181 ($p<0.001$). All consistent with primary findings.
**DISCUSSION**				
**Key Results**	18	Summarise key results with reference to study objectives	Section 4.1	Section 4.1: Primary finding: statistically significant but modest positive association between UPF consumption and perceived stress (r=0.21, R^2=4.4%, $p<0.001$). UPF Score remained significant after adjustment (B=0.175, β=0.175, p=0.003). Gender independently predicted perceived stress (B=1.42, p=0.020) — female educators reported consistently higher stress across all UPF consumption strata. Formal interaction testing not conducted; descriptive gender differences were stable across strata (+0.34 to +0.38). The small proportion of explained variance indicates UPF is one of several determinants of perceived stress.
**Limitations**	19	Discuss limitations of the study, taking into account sources of potential bias or imprecision. Discuss both direction and magnitude of any potential bias	Section 4.5	Section 4.5 — six limitations reported: 1. CROSS-SECTIONAL DESIGN: Precludes causal inference; temporal ordering cannot be established. Bidirectionality possible. 2. CONVENIENCE SAMPLING: May have recruited more engaged, health-conscious educators. External validity limited to young, urban, private-sector teaching professionals in Pune. 3. SELF-REPORT BIAS: Both exposure and outcome self-reported. Social desirability may lead to underreporting of UPF consumption; recall bias may affect accuracy. 4. RESIDUAL CONFOUNDING: Model adjusted only for age, gender, BMI (Adj. R^2=0.071). SES, income, marital status, sleep quality, physical activity, workload, teaching experience, and mental health history not assessed. Unmeasured confounding likely accounts for substantial unexplained variance. 5. EXPOSURE MISCLASSIFICATION: UPF checklist not criterion-validated against repeated 24-hour recalls. Binary scoring does not capture frequency or quantity. 6. ABSENCE OF BIOMARKERS: No inflammatory, gut microbiome, or neuroendocrine markers collected. All mechanistic pathways discussed are theoretical constructs; not empirically evaluated.

Section/Topic	Item	Recommendation	Section Reference	Relevant Text from Manuscript
**Interpretation**	20	Give a cautious overall interpretation of results considering objectives, limitations, multiplicity of analyses, results from similar studies, and other relevant evidence	Section 4.1; Conclusion	Section 4.1 and Conclusion: 'The observed association should be interpreted cautiously given the cross-sectional design and limited explained variance (R^2=0.044).' Findings are consistent with prior systematic reviews linking UPF consumption to mental health outcomes (Lane et al., 2022; Mazloomi et al., 2023). The gender effect is consistent with epidemiological evidence on occupational stress burden in women (Heub et al., 2024). Five sensitivity analyses all yielded consistent direction, supporting robustness of primary finding.
**Generalisability**	21	Discuss the generalisability (external validity) of the study results	Section 4.5	Section 4.5: Findings applicable to young (78.5% aged 20–39 years), urban, private-sector teaching professionals in Pune, Maharashtra. Not generalisable to: rural teachers; government-funded school educators; mid-career or senior cohorts; educators in smaller Indian cities. Demographic comparison with National Sample Survey (2022–23) supports broad consistency of age and gender profile with urban private-sector teachers, lending contextual plausibility despite non-probability design.
**OTHER INFORMATION**				
**Funding**	22	Give the source of funding and the role of the funders for the present study	Funding section	Funding section: 'This research received no external funding.' No commercial, institutional, or government funding was received. Institutional support provided by Symbiosis Skills and Professional University, Pune (IRB Approval No.: SSPU/R/2025/560; Date: 21.08.25) — no role in study design, data collection, analysis, interpretation, or publication decision.

Note: Completed in accordance with the STROBE Statement for cross-sectional studies (von Elm et al., 2007, PLoS Medicine). Section references above correspond to headings in the revised manuscript in lieu of page numbers, which are subject to change upon publication. Items 6b, 14c, and 16c are not applicable to cross-sectional studies. A participant flow diagram is provided as Supplementary Figure 1.
